# Co-designing a workforce retention framework for long-term care homes

**DOI:** 10.1093/geroni/igaf122.1013

**Published:** 2025-12-31

**Authors:** Winnie Sun, Jen Calver

**Affiliations:** Ontario Tech University, Oshawa, Ontario, Canada; Ontario Tech University, Oshawa, Ontario, Canada

## Abstract

**Background:**

Despite the presence of long-standing staffing issues and evidence of turnover impacting quality of resident care, there is little guidance to assist long-term care (LTC) homes to create sustainable staffing stability and retention programs. The purpose of this study is to co-develop a workforce retention framework for LTC leaders to help guide staffing stability and retention programs in their nursing department.

**Methods:**

A modified Delphi study was used to obtain consensus of workplace factors for retention of nurses and personal support workers in LTC. Forty staff members shared their level of agreement through Delphi surveys. Drawing from the literature, 54 item statements for the first survey round were organized into six categories - work/life balance, scheduling policies and procedures, professional development and education, work conditions, quality of care, and communication.

**Results:**

Study reveals resilience level factors that acknowledge the challenges of LTC work and impact on individual health and wellbeing. It appreciates that the nature of the work environment can be unpredictable as resident care complexities increase, practice guidelines and ministry mandates changes, and the implications of the prolonged staffing issue. Continuous learning reflects the quality assurance and accountability of all practicing nurses to reflect on their current practice, identifying learning needs to actively update their knowledge and skills needed for continuing competence.

**Conclusion:**

This study has implications for comprehensive staff retention planning and program development in LTC. As demand for LTC services rises, comprehensive staffing retention programs are necessary to sustain skilled and experienced staff in the workforce.

